# Yeast display platform technology to prepare oral vaccine against lethal H7N9 virus challenge in mice

**DOI:** 10.1186/s12934-020-01316-1

**Published:** 2020-03-02

**Authors:** Han Lei, Bowen Xie, Tong Gao, Qianhong Cen, Yi Ren

**Affiliations:** College of Medicine, Southwest Jiaotong University, Chengdu, 610031 China

**Keywords:** *S.cerevisiae* EBY100/pYD5-HA, Yeast display technology, Influenza A pandemic

## Abstract

**Background:**

Existing methods for preparing influenza vaccines pose the greatest challenge against highly pandemic avian influenza H7N9 outbreak in the poultry and humans. Exploring a new strategy for manufacturing and delivering a safe and effective H7N9 vaccine is needed urgently.

**Results:**

An alternative approach is to develop an influenza H7N9 oral vaccine based on yeast display technology in a timely manner. Hemagglutinin (HA) of A/Anhui/1/2013 (AH-H7N9) is used as a model antigen and characterized its expression on the surface of *Saccharomyces cerevisiae* (*S.cerevisiae*) EBY 100. Mice administrated orally with *S.cerevisiae* EBY100/pYD5-HA produced significant titers of IgG antibody as well as significant amounts of cytokines IFN-γ and IL-4. Importantly, *S.cerevisiae* EBY100/pYD5-HA could provide effective immune protection against homologous A/Anhui/1/2013 (AH-H7N9) virus challenge.

**Conclusions:**

Our findings suggest that platform based on yeast surface technology provides an alternative approach to prepare a promising influenza H7N9 oral vaccine candidate that can significantly shorten the preparedness period and result in effective protection against influenza A pandemic.

## Background

The highly pathogenic H7N9 virus has severely affected the poultry industry and posed a serious threat to human health [1]. The most effective way to curtail pandemics is by mass vaccination [2]. Currently, there are two types of licensed vaccines against seasonal influenza in the US: subunit (split) inactivated vaccines and live attenuated influenza vaccine (LAIV) [3, 4]. Both vaccines rely on embryonated chicken eggs as substrates for production. The process of constructing a new vaccine strain based on newly circulating viruses is quite lengthy. It involves in ovo (in chicken eggs) or in vitro (in cell culture using reverse genetics techniques) reassortment between the internal genes of a donor virus such as A/PR/8/34 with the hemagglutinin (HA) and neuraminidase (NA) of the new influenza strain [5]. The candidate vaccine strains must be further selected based on their high growth capability in eggs and high yield of HA content before they can be used for production of vaccines. In this case, manufacturing problems experienced in recent years illustrate that the current methods of production are fragile in ensuing an adequate and timely supply of influenza vaccine [6]. More importantly, the egg-based technology may not be suitable to respond to a pandemic crisis. Also, due to the high pathogenicity of H7N9 strains, the conventional production would require biosafety level 3 containment facilities and take several months following the identification of new potential strains. Therefore, a strategy that can rapidly produce new influenza vaccines is needed as a priority for pandemic preparedness.

*Saccharomyces cerevisiae* (*S.cerevisiae*), a nonpathogenic yeast, is an ideal organism to express viral or tumor antigens, and is the most common host for cell surface display [7, 8]. Recently, influenza H5N1 HA has been expressed on the surface of *S.cerevisiae* by C-terminal display expression plasmid pYD1 [9]. Although detailed information is provided that the HA-presented on the surface of *S.cerevisiae* has immunogenicity in animal models, intramuscularly or intraperitoneally route would bring serious inflammation since the diameter of yeast is around 10 μm which could not be absorbed completely. As a new platform based on *S.cerevisiae* N-terminal surface display technology for H7N9 vaccine development, little is known regarding the protective immunity of *S.cerevisiae*—based vaccine by oral administration route.

To address this question, we chose HA of A/Anhui/1/2013 (AH-H7N9) as a model antigen which was regarded as the strong immunogenicity for candidate vaccine target [10], and constructed *S.cerevisiae* EBY100/pYD5-HA. Further, we investigated the immunogenicity of oral administration with *S.cerevisiae* EBY100/pYD5-HA in mice. Our data demonstrate that oral vaccination with *S.cerevisiae* EBY100/pYD5-HA in the absence of mucosal adjuvant can elicit significantly humoral and cellular immune responses, as well as significant HI titers. Most importantly, *S.cerevisiae* EBY100/pYD5-HA would be able to provide effective immune protection against homologous H7N9 virus infection. These findings clearly support that influenza oral vaccine based on *S.cerevisiae* surface display technology is likely to play an important role in preventing and controlling H7N9 outbreaks and thus may provide a feasible foundation for developing safe and effective vaccines against other avian influenza viruses.

## Methods

### Plasmids, yeast and culture conditions

The HA gene (1632 bp) of A/Anhui/1/2013 (AH-H7N9) was PCR-amplified from pCDNA3.1/H7N9/HA using the following primers: HA-F: CTAGCTAGCAATGCAGACAAAATC (*Nhe* I); HA-R: CCGGAATTCTATACAAATAGTGCACC (EcoRI) and subcloned into the yeast display plasmid, pYD5, which was kindly provided by Dr. Z Wang [11] and allowed the NH_2_ terminus of the displayed protein of interest to be free. The shuttle plasmid pYD5-HA was transformed into competent *Escherichia coli* DH5α (New England Biolabs, Beverly, MA) and then electroporated into competent *S.cerevisiae* EBY100 (Invitrogen, San Diego, CA). Recombinant yeast transformants were grown on selective plate which contained 0.67% yeast nitrogen base (YNB) without amino acids, 2% dextrose, 0.01% leucine, 2% agar and 1 M sorbitol at 30 °C for 3 days. Single positive clone *S.cerevisiae* EBY100/pYD5-HA was selected and cultured in 3 mL of YNB-CAA (20 g/L dextrose, 6.7 g/L yeast nitrogen base without amino acids, 13.61 g/L Na_2_HPO_4_, 7.48 g/L NaH_2_PO_4_ and 5 g/L casamino acids) overnight at 30 °C with shaking. Inducible expression of *S.cerevisiae* EBY100/pYD5-HA was performed in YNB-CAA medium where dextrose was replaced by 20 g/L of galactose at 20 °C for 3 days with shaking. Meanwhile, *S.cerevisiae* EBY100 containing empty pYD5 was used as a negative control for the following tests.

### Detection of HA protein expression

1 OD_600nm_ of *S.cerevisiae* EBY100/pYD5-HA pellets (1 OD_600nm_ ≈ 10^7^ cells) was collected at 72 h post-induction, and washed three times with 500 µL of sterile phosphate-buffered saline (PBS) for Western blotting, immunofluorescence and flow cytometric assay.

For Western blot analysis, 1 OD_600nm_ of *S.cerevisiae* EBY100/pYD5-HA pellets were re-suspended with 50 µl of 6× loading buffer and boiled for 10 min. Treated samples were resolved using SDS–polyacrylamide gel electrophoresis and then electrophoretically transferred to nitrocellulose membrane (Bio-rad, Hercules, California, USA). After blocking with 5% non-fat milk at room temperature for 2 h, the blot was probed with a monoclonal mouse anti-HA antibody (1: 500 diluted) (kindly provided by NIH Biodefense and Emerging Infections Research Resources Repository, Manassas, VA, USA), and incubated overnight at 4 °C. The membrane was followed by 1: 5000 diluted horseradish peroxidase (HRP)-conjugated anti-mouse IgG (Sigma-Aldrich Corporation, St. Louis, MO, USA) at room temperature for 1 h and developed with the West Pico Chemiluminescent Substrate (Thermo Fisher Scientific Inc., Rockford, IL, USA) and imaged using Molecular Imager ChemiDoc XRS System (Bio-Rad Laboratories, Inc., Hercules, CA, USA). Furthermore, 1 OD_600_ of *S.cerevisiae* EBY100/pYD5-HA pellets were treated by PNGase F kit (New England Biolabs, Beverly, MA, USA) for detecting N-glycosylation of HA protein.

For immunofluorescence assay and flow cytometric analysis, 1 OD_600nm_ of *S.cerevisiae* EBY 100 pellets was incubated with a monoclonal mouse anti-HA antibody (1: 500 diluted) at 4 °C for 1 h, and followed by FITC-conjugated goat anti-mouse IgG (1: 5000 diluted) at 4 °C for 30 min and re-suspended with 500 µL of sterile PBS. Finally, 5 µL of *S.cerevisiae* EBY100/pYD5-HA pellets were used for immunofluorescence assay (Olympus IX70, Japan), and 300 µL of *S.cerevisiae* EBY100/pYD5-HA cells were analyzed by flow cytometry analysis (BD FacsCalibur, BD Bioscience, San Jose, CA, USA), respectively.

### Quantification of *S.cerevisiae* EBY100/pYD5-HA expressing HA protein by indirect ELISA

*S.cerevisiae* EBY100/pYD5-HA expressing HA protein was assayed using a modified, previously published ELISA protocol [12]. Briefly, 10 OD_600nm_ of *S.cerevisiae* EBY100/pYD5-HA cells (1 OD_600nm_ ≈ 10^7^ cells) were re-suspended in 100 μL of a monoclonal mouse anti-HA antibody (0, 5, 10, 25, 50, 75, 100, 125 and 150 μg/mL) (kindly provided by NIH Biodefense and Emerging Infections Research Resources Repository, Manassas, VA, USA) in PBS containing 1% BSA and incubated at room temperature for 1 h. Then, cells were washed 3 times in PBS. Goat anti-mouse IgG antibody conjugated with horseradish peroxidase (1 mg/ml) (Sigma-Aldrich Corporation, St. Louis, MO, USA) was added and the mixture was incubated at room temperature for 1 h. After washing in the same way, the cells were re-suspended in 100 μL of PBS and subjected to protein surface detection by incubating in 100 μl of HRP substrate 3,3′,5,5′-tetramethylbenzidine (TMB) (Sigma-Aldrich Corporation, St. Louis, MO, USA) in the dark for 30 min followed by addition of 100 μl of 2 mol/L H_2_SO_4_ to stop the reaction. The yeast cell suspension was spun down and the supernatant OD_450_ was measured using a microplate reader. *S.cerevisiae* EBY100/pYD5 was used a negative control.

### Vaccine, animals, immunization and virus challenge

*S.cerevisiae* EBY100/pYD5-HA cells were harvested at 72 h post-induction and treated by heat-inactivation at 60 °C for 1 h. The final concentration of *S.cerevisiae* EBY100/pYD5-HA was adjusted to 1.0 OD_600nm_/µL using sterile PBS and stored at 4 °C until use.

Eight-week-old female BALB/c mice (Jackson Laboratories, ME, USA) were used for all studies and housed in the specific pathogen-free (SPF) facilities. Mice (n = 10/group) were vaccinated orally with 150 OD_600nm_ of *S.cerevisiae* EBY100/pYD5-HA on day 1 for prime immunization and boosted on day 14. For comparison, 150 OD_600nm_ of *S.cerevisiae* EBY 100/pYD5 or 150 μL of PBS was used as a negative control. Mice weights were recorded at pre-immunization and post-immunization.

For the virus challenge experiments, all vaccinated mice were anesthetized and intranasally inoculated with 50 μL of 10 × 50% lethal dose (LD_50_) A/Anhui/1/2013 (AH-H7N9) virus on day 35 after the initial immunization. Mice were monitored for survival and body weight change for 14 days. At day 3 post infection, mice (n = 3/group) were humanely euthanized, lungs were collected and washed with 1.0 mL of sterile PBS. A TCID_50_ assay was determined virus titers from clarified homogenates [9]. All animal immunizations complied with the Guidelines for Use and Care of Experimental Animal s and were approved by the Animal Committee of the Institute of Southwest Jiaotong University.

All virus challenge experiments with A/Anhui/1/2013 (AH-H7N9) were performed under enhanced biosafety level-3(BLS3)-plus containment facilities.

### Enzyme-linked immunosorbent assay (ELISA)

Sera were isolated from blood samples collected at day 13 and 28 after the initial immunization, and stored at − 20 °C until use. Avidin–biotin system (ABS)-enzyme-linked immunosorbent assay (ELISA) was conducted to measure HA-specific IgG titers, as described previously [13]. Briefly, 96-well microplates (Costar, Corning, NY, USA) were coated with 2 μg/mL of recombinant HA protein, and incubated overnight at 4 °C. Plates were washed three times with TBS containing 0.05% Tween 20 (TBST) and then blocked with TBST containing 1% bovine serum albumin (BSA) at room temperature for 2 h. twofold diluted sera samples were added and incubated at 37 °C for 1 h. Bound antibodies were detected using biotinylated goat anti-mouse IgG (1: 5000 diluted) and followed by 1: 1000 diluted alkaline phosphatase-conjugated streptavidin (R&D Systems, USA). Finally, plates were developed using a pNPP phosphatase substrate (MP Biomedicals, Santa Ana, CA, USA), and the reaction was allowed to develop at room temperature for 25 min and then stopped by adding 50 μL of 2 mol/L NaOH to each well. Optical density (OD) was measured at 405 nm. The IgG titer was determined to be the lowest serum dilution with an OD greater than twice the mean OD of naïve serum plus 2 standard deviations.

### ELISpot assay

Capture antibodies mouse IFN-γ and IL-4 cytokines (3 µg/mL in coating buffer, R&D Systems, USA) were coated on the Multiscreen 96-well filtration plates (Millipore, Billerica, MA, USA), respectively. Spleen cells (n = 3/group) were harvested at 2 weeks post prime-boost immunization. Freshly isolated splenocytes (1 × 10^6^ cells) were cultured on the plate with 100 µL of RPMI 1640 media with 10% FBS, and then stimulated with or without 10 μg/mL of HA-specific peptide (PKGRGLFGAIAGFIENGWEGL) for 36 h in a humidified 37 °C CO_2_ incubator. After incubation, the plates were washed with sterile PBS and further incubated with biotinylated anti-mouse IFN-γ and IL-4 antibodies (1:5000 diluted). After three additional washes, alkaline phosphatase (AP) conjugated streptavidin (1: 1000) was added to each well and incubated at room temperature for 3 h. The plates were washed and developed with BCIP/NBT Chromogen (R&D Systems, USA). The number of IFN-γ and IL-4 secreting cells was counted using an ImmunoSpot ELISpot plate reader (Cellular Technology Ltd, Shaker Heights, OH, USA).

### Hemagglutination inhibition (HI) assay

HI assay was performed to assess specific response to HA of H7N9 virus, as described previously [13]. Briefly, sera were treated by Receptor destroying enzyme (RDE) (Denka-Seiken, Tokyo, Japan) at 37 °C overnight, and then followed by heat-activation at 56 °C for 45 min. Two fold serial diluted samples (25 μL) in V-bottom 96-well plates were mixed with the equal volume of 4 HA units each well and incubated at room temperature for 30 min. 50 μL of 0.5% (v/v) chicken red blood cells (cRBCs) was added to each well, and hemagglutination was assessed visually after 40 min. The HI titer was expressed as the reciprocal of the highest dilution of the samples and the significant HI titer was greater than 16.

### Statistical analysis

A two-tailed Student’s t-test and one-way ANOVA with post hoc analysis were used when comparing two different groups. A *p* value less than 0.05 was considered to be significant.

## Results

### Expression of HA protein on the surface of *S.cerevisiae* EBY 100

**Fig. 1:**
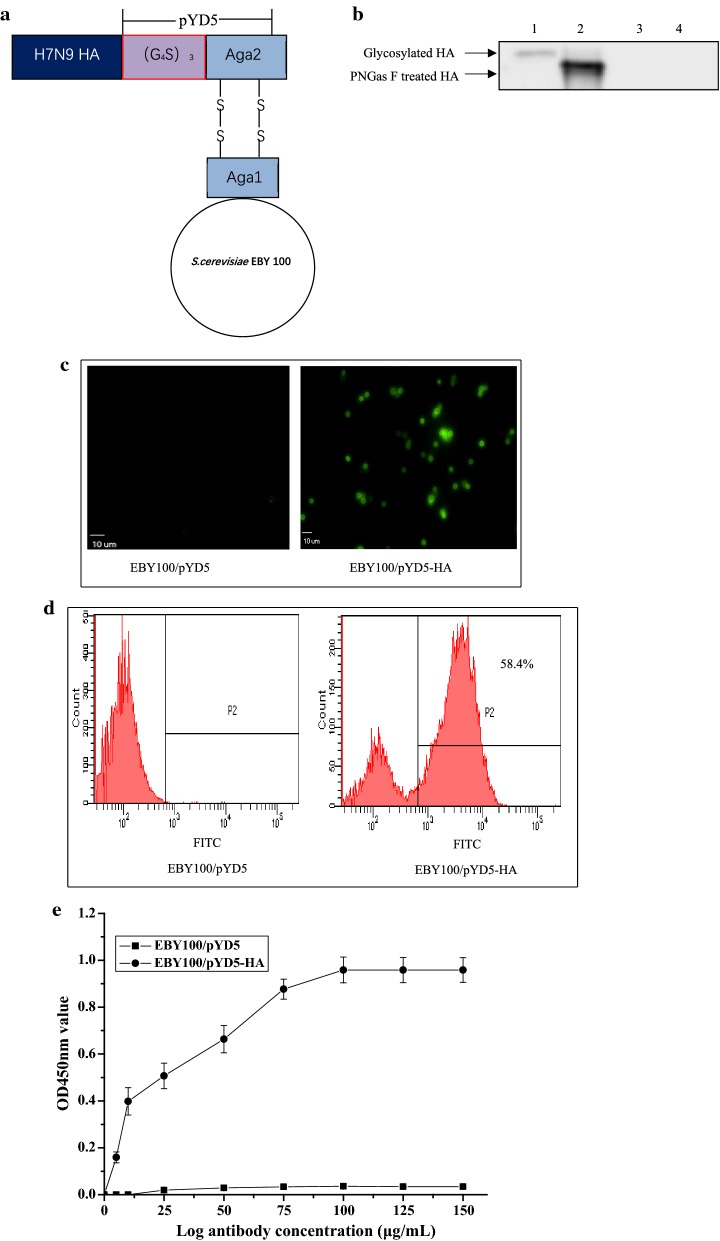
Construction of yeast display vector and analysis of the expression of HA protein. **a** Schematic diagram of *S.cerevisiae* EBY100/pYD5-HA. The HA-Aga2 fusion protein binds to Aga1 through two disulfide bonds after its secretion from the *S*.*cerevisiae*. Aga1 is the first subunit of the yeast **a**-agglutinin receptor. A GS linker is inserted between Aga2 and HA to stabilize the fusion protein expression. **b** Western blotting analysis. Lane 1: HA glycoprotein. Lane 2: Deglycosylation of HA protein. Lane 3: *S.cerevisiae* EBY100/pYD5 control. Lane 4: Deglycosylation of *S.cerevisiae* EBY100/pYD5. **c** Immunofluorescence microscope. *S.cerevisiae* EBY100/pYD5 (left) and *S.cerevisiae* EBY100/pYD5-HA (right) (magnification 400×). **d** Flow cytometric analysis. *S.cerevisiae* EBY100/pYD5 (left) and *S.cerevisiae* EBY100/pYD5-HA (right). **e** Quantification of *S.cerevisiae* EBY100/pYD5-HA expressing HA protein by indirect ELISA. The values were obtained from three independent experiments. Bar = mean ± SD

To determine the functional display of HA on yeast surface, the HA gene without signal peptide was subcloned into a display plasmid pYD5 led to the fusion of HA to N—terminus of Aga2, the second subunit of **a**-agglutinin receptor (Fig. 1a). The C-terminus of Aga2 entails both a secretion signal peptide and a binding site for Aga1, another subunit of the yeast **a**-agglutinin. The Aga2-HA was bound to Aga1 through two disulfide bonds, resulting in the HA display on yeast wall. The Aga1 gene was pre-integrated into the chromosome of the yeast.

Expression of HA protein was confirmed by Western blotting (Fig. 1b). As we expected, a specific band was observed at expected size for HA glycoprotein (approximately 65 kDa) (Fig. 1b, Lane 1).

On the other hand, to determine whether HA protein has a functional N-glycosylation, *S.cerevisiae* EBY 100/pYD5-HA cells were treated by PNGase F kit for detection of deglycosylation. As shown in Fig. 1b Lane 2, an expected size was indicated that HA protein had N-glycosylation modification. As a result, no specific bands were observed in *S.cerevisiae* EBY 100/pYD5 control cells. Overall, these findings indicate HA protein possess the post translational process of N-glycosylation.

Further, we performed immunofluorescence assay and flow cytometric analysis. As compared to *S.cerevisiae* EBY100/pYD5 controls, strong fluorescence derived from *S.cerevisiae* EBY100/pYD5-HA was observed (Fig. 1c, d). Taken together, these results demonstrate that HA protein display on the surface of *S.cerevisiae* EBY100 can be recognized by a monoclonal mouse anti-HA antibody that showing high binding specificity.

### Quantification of HA protein on the yeast surface by indirect ELISA

When increasing concentrations of monoclonal anti-HA antibody was used against 10 OD_600nm_ of *S.cerevisiae* EBY100/pYD5-HA cells/mL expressing HA, it was found that yeast displayed HA at approximately 100 μg on the cell surface (Fig. 1e). When the concentration of antibodies was increased beyond this point, the optical densities were more or less stable suggesting that expression of HA proteins on the cell surface was at its saturation limit at 100 μg/mL. In other words, 10 OD_600nm_ of *S.cerevisiae* EBY100/pYD5-HA had 100 μg of HA protein displayed on the yeast surface, which was 1 OD_600nm_ of *S.cerevisiae* EBY100/pYD5-HA had 10 μg of HA protein. This yeast-ELISA method was a high-throughput one to detect surface proteins without the need for protein extraction and purification [14].

Mice weights were recorded at pre-immunization and post-immunization, as shown in Table 1. These data indicate that mice vaccinated orally with recombinant yeast have no significant weight changes for prime-boost immunization.

**Table 1:** Mice weighs change at pre- and post-immunization (n = 10/group)

Group	Dose	Prime		Boost	
		Pre-immunization	Post-immunization	Pre-immunization	Post-immunization
PBS	150 μL	20.52 ± 1.21^*^	20.52 ± 1.21^*^	20.61 ± 1.21^*^	20.61 ± 1.21^*^
EBY100/pYD5	150 OD_600nm_	20.58 ± 1.11^*^	20.52 ± 1.11^*^	20.54 ± 1.11^*^	20.52 ± 1.11^*^
EBY100-pYD-HA	150 OD_600nm_	20.56 ± 1.12^*^	20.50 ± 1.11^*^	20.51 ± 1.11^*^	20.50 ± 1.11^*^

The data are presented as mean ± standard deviation (SD)

Asterisk indicates statistical significance compared to *S.cerevisiae* EBY100/pYD5 and PBS groups (*p *< 0.05)

### Antibody response and HI assay

**Fig. 2:**
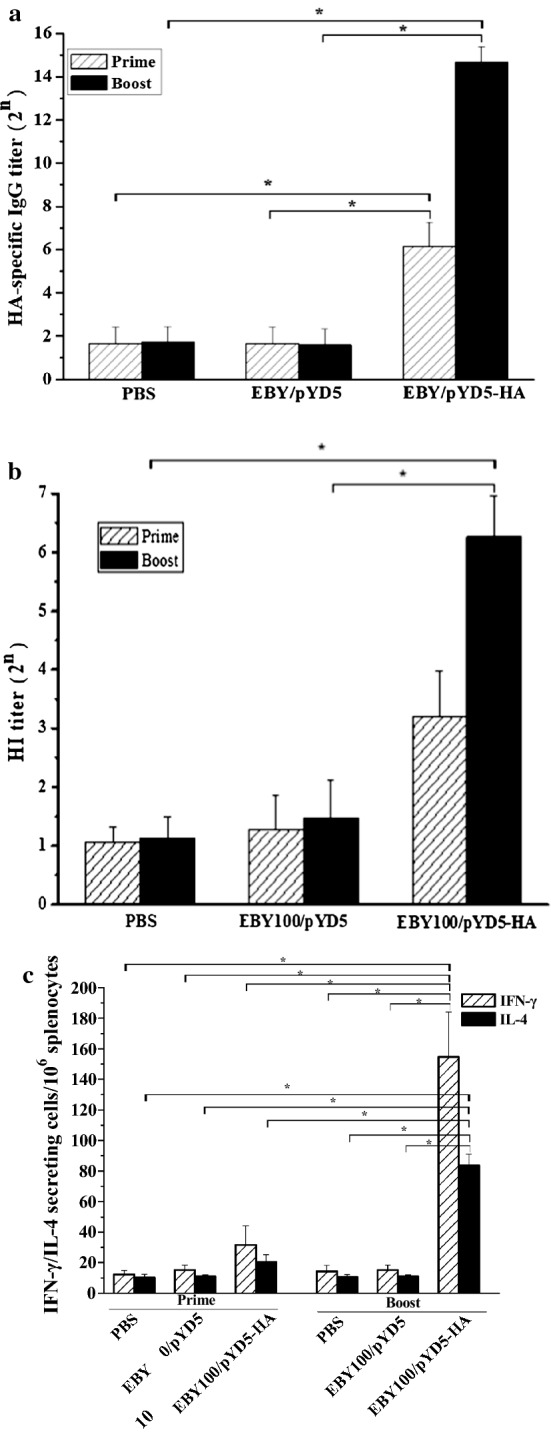
Antibody response detected by ELISA and Hemagglutination inhibition (HI) assay. **a** HA - specific IgG titers. **b** HI titers. **c** The cellular immune responses were assayed by ELISpot assay. Splenocytes derived from vaccination (n = 3/group) were incubated on the IFN-γ or IL-4 capture antibody coated with stimulation of HA peptide. IFN-γ and IL-4 spots were counted. The data are presented as mean ± standard deviation (SD). Asterisk indicates statistical significance compared to *S.cerevisiae* EBY100/pYD5 and PBS groups (*p *< 0.05)

The antibody responses in the sera of mice vaccinated orally with *S.cerevisiae* EBY100/pYD5-HA were evaluated by ELISA. Mice that received *S.cerevisiae* EBY100/pYD5-HA produced a low and detectable level of HA- specific IgG antibody after the prime immunization, and greatly increased to a high level after boost immunization (Fig. 2a). In contrast, no HA-specific antibodies were observed in PBS or *S.cerevisiae* EBY100/pYD5 group (Fig. 2a). Collectively, these results highlight a prime-boost immunization regimen with *S.cerevisiae* EBY100/pYD5-HA can induce strong humoral immune responses in a mouse model.

Further, we assessed the functional significance of the antibody responses induced by *S.cerevisiae* EBY100/pYD5-HA, hemagglutinin inhibition (HI) titers were determined by measuring the ability of sera to inhibit agglutination of chicken erythrocytes. As compared to the control groups (PBS or *S.cerevisiae* EBY100/pYD5), significant HI titers were observed at higher levels after boost immunization (Fig. 2b). Therefore, unadjuvanted *S.cerevisiae* EBY100/pYD5-HA was immunogenic and could induce higher levels of HI titers which might have a correlation with immune protection.

### Cellular immune responses induced by *S.cerevisiae* EBY100/pYD5-HA

To investigate the cellular immune responses induced by *S.cerevisiae* EBY100/pYD5-HA, we determined IFN-γ and IL-4 secreting splenocytes by ELISpot. Splenocytes were isolated at 2 weeks post prime-boost immunization. After stimulation with HA specific peptide, higher levels of IFN-γ and IL-4 secreting cells were observed in the *S.cerevisiae* EBY100/pYD5-HA group than those in the control groups that received PBS or *S.cerevisiae* EBY100/pYD5 (Fig. 2c). However, the levels of IFN-γ secreting splenocytes were significant higher than IL-4 levels in the mice vaccinated orally with *S.cerevisiae* EBY100/pYD5-HA (Fig. 2c). Taken together, *S.cerevisiae* EBY100/pYD5-HA could induce Th1 and Th2 type immune responses with preferences of the Th1 type immune responses as evidenced by higher levels of IFN-γ production.

### Protective immunity induced by *S.cerevisiae* EBY100/pYD5-HA

**Fig. 3:**
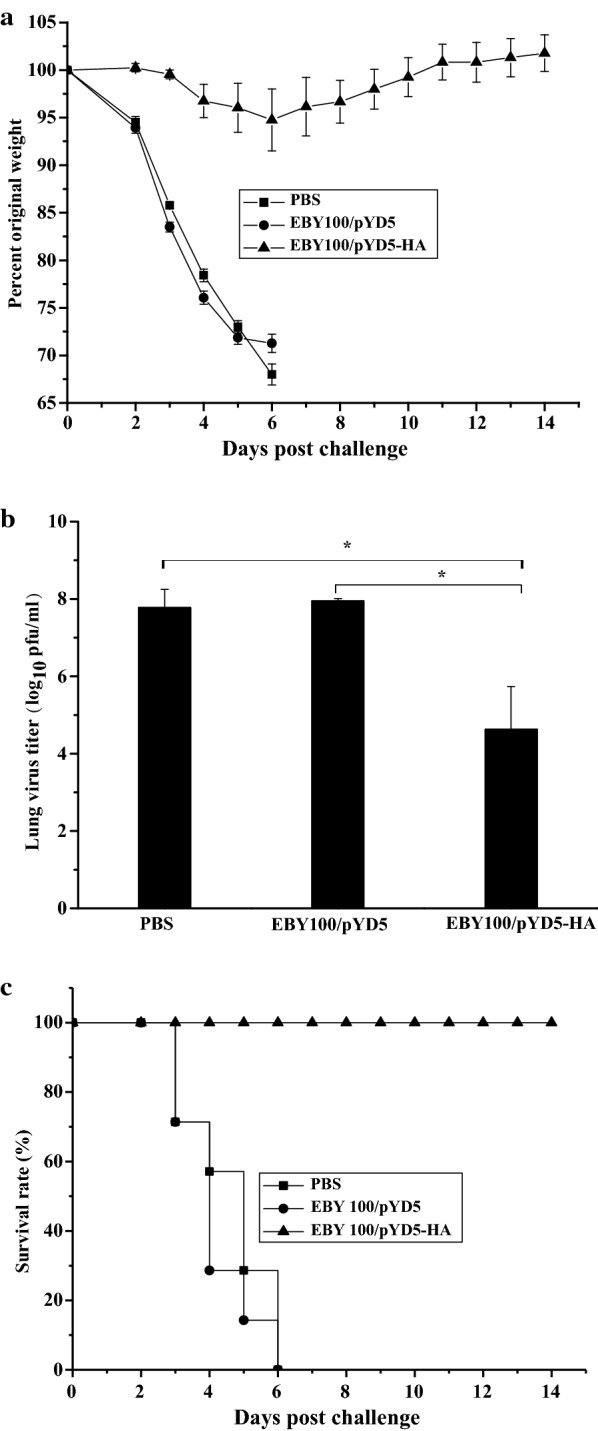
Immune protection conferred by *S.cerevisiae* EBY100/pYD5-HA against lethal H7N9 virus challenge. Mice were intranasally challenged with a lethal dose (10 × LD_50_) of A/Anhui/1/2013 (AH-H7N9) virus at 2 weeks after the final immunization (n = 10/group). **a** Weight change as a percentage. Bars indicate SDs. **b** Lung viral titers were determined by a plaque assay at day 3 after challenge (n = 3 of 10 challenged mice). **c** Survival rate. Asterisk indicates significant difference compared to *S.cerevisiae* EBY100/pYD5 and PBS groups (*p *< 0.05)

To determine whether *S.cerevisiae* EBY100/pYD5-HA could elicit protection against lethal homologous H7N9 virus infection, immunized mice were intranasally challenged with 50 μL 10 × LD_50_ of A/Anhui/1/2013 (AH-H7N9) virus at 2 weeks after the final immunization, and their health status was monitored for 14 days. As shown in Fig. 3, the control groups that received PBS or *S.cerevisiae* EBY100/pYD5 showed clinical signs of severe disease and significant body weight loss staring on day 3 after virus infection, and died or reached the humane euthanasia endpoint on 6 days post challenge. In contrast, all mice vaccinated with *S.cerevisiae* EBY100/pYD5-HA were 100% protected from lethal challenge and no significant body weight loss was observed (Fig. 3a, b).

We also determined virus titers in the lungs of the challenged mice (n = 3) on day 3 post infection. The lung titers of mice vaccinated with *S.cerevisiae* EBY100/pYD5-HA were approximately 600 fold lower than the naïve control (PBS) and the negative control (*S.cerevisiae* EBY100/pYD5). Thus, our results show that oral immunization with *S.cerevisiae* EBY100/pYD5-HA could decrease virus shedding in the lungs.

Taken together, *S.cerevisiae* EBY100/pYD5-HA can provide effective protection against H7N9 virus infection. These results further support that this platform based on yeast surface display technology can provide a feasible strategy for developing safe and effective influenza oral vaccines.

## Discussion

Highly pathogenic avian influenza (HPAI) H7N9 virus remains global concerns. Current strategies for developing and manufacturing these vaccines are time consuming and somewhat limited in the ability to generate vaccines quickly [15]. Furthermore, conventional influenza vaccines utilizing the HA and NA of influenza viruses have safety and production issues [16]. To address these questions, we hypothesized that a new platform for manufacturing influenza vaccines based on yeast surface display technology could provide potential of protective immunity. In the present study, HA was chosen as a model antigen for influenza vaccine development because of that HA of influenza H7N9 virus has strong immunogenicity and neutralizing activity [1, 6, 17]. Thus, we generated HA protein of A/Anhui/1/2013 (AH-H7N9) virus was displayed on the surface of *S.cerevisiae* EBY100 (Fig. 1a). Further, we tested its immunogenicity in mice by oral administration without the presence of mucosal adjuvant. Notably, HA presented on the surface of *S.cerevisiae* EBY100 could not only elicit strongly humoral immune response, as well as significant cellular immune response, but also confer effective protection against H7N9 virus. Therefore, this study provides the first evidence supporting an alternative strategy for developing influenza pandemic oral vaccines based on yeast surface display technology.

The cell surface display technology can be designed to express a target on the surface of cell through linkage with a genetically fused anchor protein. Compared to intracellular expression of viral proteins, the display of viral antigen on cell surface or wall can facilitate their recognition by host immune system, thereby enhancing their capability of eliciting protective immunity in the vaccinated hosts [18]. Basically, there have N-terminal (target protein-anchor protein fusion) and C-terminal fusion (anchor protein-target protein fusion) methods for *S. cerevisiae* surface displays [7]. In this study, the N-terminus of the anchor protein (Aga2p) is genetically fused to the C-terminus of HA protein via Glycine–Serine linker, resulting in free N-terminal display of HA (Fig. 1a). To elevate the HA display on yeast surface, the culture temperature was lowered from 30 °C to 20 °C after galactose-induction. Further, recombinant *S.cerevisiae* EBY100/pYD5-HA was confirmed for high display efficiency of HA protein (Fig. 1c, d) and quantification of HA protein on the yeast surface was determined by indirect ELISA (Fig. 1e) which would produce sufficient antigens for subsequent oral vaccination. These findings clearly indicate HA can be presented on the surface of *S.cerevisiae* EBY100 with stable and high expression efficiency which has great contributions to immune efficacy by oral administration route.

One of the reasons for interest in recombinant *S.cerevisiae* as a vaccine vehicle is its lacking of toxicity [8]. Besides being inherently nonpathogenic, this particular species of yeast can be heat-killed before administration and has been shown to be safe in humans in several clinical trials, with maximum tolerated dose not reached [19, 20]. Most importantly, recombinant *S. cerevisiae* has been shown to induce a strong host immune response to non-self-antigens [21–23]. In addition to the convenience of production, for purposes of vaccination, yeast has been shown to have natural adjuvant activity making the expressed proteins more immunogenic when administered along with yeast cell wall components [8]. Development of genetic systems to display foreign proteins on the surface of yeast via fusion to glycosylphosphatidylinositol-anchored (GPI) proteins has further simplified the purification of recombinant proteins by not requiring harsh treatments for cellular lysis or protein purification [24]. These characteristics make *S. cerevisiae* a potential tool for vaccine delivery.

Our team has constructed *S.cerevisiae* EBY100/pYD1-HA which C terminal of HA is free and then investigated its immunogenicity by conventional injection immune route (i.m. or i.p.) [9]. However, due to the diameter of *S.cerevisiae* is around 10 μm, oral administration is more safe and effective immune route than injectable route for yeast surface display system. Consequently, we firstly tested the immunogenicity of *S.cerevisiae* EBY100/pYD5-HA by oral vaccination.

Current influenza vaccines based on conventional manufacturing platform have failed to provide sufficient protection against infections of rapidly mutated influenza viruses. Thus, a novel platform based on surface display technology of *S. cerevisiae* can meet requirements of developing safe and effective influenza oral vaccines. The main goal of this study was to evaluate the immune efficacy and protective immunity based on *S. cerevisiae* surface display technology by oral administration route. HI antibody response is an important factor for evaluating immunogenicity against corresponding viruses. Significant HI titer was obtained in the *S.cerevisiae* EBY100/pYD5-HA (Fig. 2b). Regarding the T cell responses, mice vaccinated with *S.cerevisiae* EBY100/pYD5-HA were found to be more effective in generating T cells secreting IFN-γ indicating Th1 responses, as compared to T cells secreting IL-4 cytokine representing Th2 type responses, which was not a protective response (Fig. 2c). All mice that received *S.cerevisiae* EBY100/pYD5-HA group were completely protected from lethal challenge of H7N9 virus (Fig. 3c), and remained healthy and showed no signs of abnormal behavior after virus infection. Collectively, these findings reinforced how significant it is use *S. cerevisiae* display system for developing safe and effective influenza vaccine against H7N9 virus.

In conclusion, this study represents a new platform based on *S.cerevisiae* surface display technique for manufacturing influenza vaccine using HA as a model antigen for protection against highly pathogenic strains of avian influenza. Considering the current platforms for approved influenza vaccines, this platform will offer significant advantages for influenza oral vaccine development particularly if a pandemic occurs. Future studies will be needed to elucidate the mechanism of the cross-protective immunity induced by the proposed candidate vaccines, and investigate detailed comparative immunogenicity and protective of yeast vaccines in comparison with inactivated whole virus and attenuated live H7N9 vaccines.

## Conclusions

This study describes the construction of a new yeast display platform for presentation of virus antigen that H7N9 HA is used as a model. Based on the new design method for virus antigen display on the surface of yeast, NH_2_ terminus of HA is free. Further, the high immunogenicity is investigated by oral administration route in mice. The obtained data strongly suggest that N-terminal yeast display platform can be considered for virus or bacteria oral vaccine development.

## Data Availability

The datasets generated and analyzed during the current study are available from the corresponding author on reasonable request.
